# Definitions in Laser Technology

**DOI:** 10.4103/0974-2077.53103

**Published:** 2009

**Authors:** Rabindra Kumar Yadav

**Affiliations:** *Trainee Dermatologist, Venkat Charmalaya—Centre for Advanced Dermatology, Bangalore, Karnataka, India.*

**Laser:** An instrument that generates a beam of light of a single wavelength or color that is both highly collimated and coherent; an acronym that stands for light amplification by the stimulated emission of radiation

**Laser medium:** A material or substance of solid, liquid, or gaseous nature that is capable of producing laser light due to stimulated electron transition from an unstable high-energy orbit to a lower one with release of collimated, coherent, monochromatic light

**Pump:** The electrical, optical, radiofrequency or chemical excitation that provides energy to the laser medium

**Optically pumped laser:** A laser where electrons are excited by the absorption of light energy from an external source

**Electromagnetic radiation:** A complex system of radiant energy composed of waves and energy bundles that is organized according to the length of the propagating wave

**Photon:** A quantum of electromagnetic radiation or light

**Monochromatic:** Light energy emitted from a laser optical cavity of only a single wavelength

**Coherence:** All waves are in phase with one another in both time and space

**Collimation:** All waves are parallel to one another with little divergence or convergence

**Population inversion:** The state present within the laser optical cavity (resonator) where more atoms exist in unstable high-energy levels than their normal resting energy levels

**Power:** The rate at which energy is emitted from a laser

**Power density (irradiance):** The quotient of incident laser power on a unit surface area, expressed as watts/cm^2^

**Energy fluence:** The energy contained within light is expressed in joules (J). The *energy fluence* determines the amount of laser energy delivered in a single pulse and is expressed in joules/cm^2^

**Pulse:** The brief span of time for which, the focused and scanned laser beam interacts with a given point on the skin (usually in milliseconds, nanoseconds)

**Q-Switch:** An optical device (Pockels cell) that controls the storage or release of laser energy from a laser optical cavity. Q-switching is a means of creating very short pulses (5-100 ns) with extremely high peak powers. Q stands for quality.

**Quality factor (Q factor)** is defined as the ratio of the energy stored in the optical resonant cavity to the energy loss per cycle. The higher the quality factor, the lower the losses. In the technique of Q-switching, energy is stored in the amplifying medium by optical pumping while the cavity Q is lowered to prevent the onset of laser emission. When a high cavity Q is restored, the stored energy is suddenly released in the form of a very short pulse of light. Q-switched lasers are often used in applications which demand high laser intensities in nanosecond pulses

**Reflectance:** The ratio of incident power to absorbed power by a given medium

**Scattering:** A change in the direction of propagation of a photon. This results from imprecise absorption of laser energy by a biologic system resulting in a diffuse effect on tissue

**Absorption:** The transformation of radiant energy to another form of energy (usually heat) by interacting with matter

**Chromophore:** Endogenous light-absorbing chemicals, which absorb light of specific wavelength

**Transmission:** The passage of laser energy through a biologic tissue without producing any effect

**Thermomodulation:** The ability of low-energy light to upregulate certain cellular biologic activities without producing an injury

**Photoacoustic effect:** The ability of Q-switched laser light to generate a rapidly moving wave within living tissue that destroys melanin pigment and tattoo ink particles

**Selective photothermolysis:** A concept used to localize thermal injury to a specific target based on its absorption characteristics, the wavelength of light used, the duration of the pulse, and the amount of energy delivered

**Extended theory of selective theromolysis:** This distinguishes between an ‘absorber’ chromophore (e.g. melanin in hair shaft) in which heat is generated and a distant target (e.g. stem cells of isthmus), to which heat is transmitted and which is damaged as a result.

**Fractional photothermolysis:** Here, pinpoint laser pulses create thousands of microthermal zones (MTZ), which are microscopic epidermal and dermal thermal wounds interspersed within untreated tissue. The small wound size and short migratory distance for keratinocytes facilitate rapid epidermal repair resulting in effective skin rejuvenation and quick recovery

**Thermal relaxation time:** It is the time taken for the target to dissipate about 63% of the incident thermal energy. It is related to size of target chromophore, e.g., few nanoseconds (tattoo particles) to hundred milliseconds (leg venules)

**Thermal damage time:** It is the time required, for the entire target, including the primary chromophore (e.g. melanin) and the surrounding target (e.g. hair follicle), to cool by about 63%. It includes cooling of the primary chromophore as well as the entire target.

**Extinction length:** The thickness of material necessary to absorb 98% of incident energy

**Nd:YAG:Neodymium:** Yttrium Aluminum Garnet, (YAG) a widely used solid-state crystal composed of yttrium and aluminum oxides and a small amount of the rare earth neodymium.

## References

[1] Tanzi EL, Lupton JR, Alster TS (2003). Lasers in dermatology: Four decades of progress. J Am Acad Dermatol.

[2] Watanabe S (2008). Basics of laser application to dermatology. Arch Dermatol Res.

[3] Goldberg DJ (2007). Procedures in Cosmetic Dermatology Series. Lasers and Lights.

[4] Boixeda P, Calvo M, Bagazgoitia L (2008). Recent Advances in Laser Therapy and Other Technologies. Actas Dermosifiliogr.

[5] Carroll L, Humphreys TR (2006). LASER-tissue interactions. Clin Dermatol.

[6] Goldberg DJ (2005). Laser Dermatology.

